# Risk Assessment of Dietary Exposure to Fluoride from Follow-On Milk Consumption

**DOI:** 10.3390/foods14213728

**Published:** 2025-10-30

**Authors:** Santiago Cerdán-Pérez, Soraya Paz-Montelongo, Samuel Alejandro-Vega, Carmen Rubio, Ángel J. Gutiérrez, Arturo Hardisson, Chaxiraxi de la Cruz Morales, Inés A. Revelo-Mejía, Javier Darias-Rosales, Natalia Pérez-Rodríguez, Consuelo Revert

**Affiliations:** 1 Area of Toxicology, Universidad de La Laguna, 38071 La Laguna, Tenerife, Spainsalejand@ull.edu.es (S.A.-V.); crubio@ull.edu.es (C.R.);; 2 Programa de Doctorado en Ciencias Médicas y Farmacéuticas, Desarrollo y Calidad de Vida, Universidad de La Laguna, 38071 La Laguna, Tenerife, Spain; 3 Grupo Interuniversitario de Toxicología Ambiental y Seguridad de los Alimentos y Medicamentos, Universidad de La Laguna, 38071 La Laguna, Tenerife, Spain; 4 Area of Pharmacology, Universidad de La Laguna, 38071 La Laguna, Tenerife, Spain; 5 Facultad de Odontología, Universidad Antonio Nariño, Bogotá 110231, Colombia; 6 Oncologia Médica, Hospital Universitario Nuestra Señora de Candelaria, 38010 Santa Cruz de Tenerife, Tenerife, Spain; 7 Area of Oncology, Universidad de La Laguna, 38071 La Laguna, Tenerife, Spain; 8 Departamento de Medicina Física y Farmacología, Universidad de La Laguna, 38071 La Laguna, Tenerife, Spain

**Keywords:** fluoride, follow-on milk, exposure assessment, fluorosis, toxicity

## Abstract

Breastfeeding based on the use of follow-on milk may contain traces of xenobiotic elements that could pose a risk to the health of the vulnerable population for which it is intended. Fluorine is a non-essential element that, at high concentrations, can produce adverse health effects such as dental fluorosis, decreased IQ (intelligence quotient), thyroid alterations, and kidney damage. Given the vulnerability of infants and the possible presence of fluoride in this type of product, the content of this anion was determined in a total of 46 samples of follow-on milk from different brands and types (starter, follow-on, and hydrolysate formulas) using a fluoride ion-selective electrode (EWI). The highest mean concentration of fluoride was recorded in the hydrolysate formulas (3.38 ± 2.78 mg/L). The dietary intake assessment indicated that some brands of hydrolyzed formulas could pose a health risk, providing up to 94.1% of the UL (upper level) with only one 90 mL serving in the 0–6-month age group. It is recommended that consumers be aware of the fluoride content in the water used to prepare bottles, as it can further increase total fluoride intake and therefore pose a risk to the health of infants.

## 1. Introduction

The World Health Organization (WHO) and numerous scientific societies recommend exclusive breastfeeding for the first 6 months of age and, subsequently, the introduction of complementary feeding [1,2,3]. Extensive research has demonstrated the benefits of this practice for newborns, such as the maturation of the central nervous and immune systems, the establishment of psycho-affective bonds between mother and child, decreased cardiovascular risk, infections, increased proinsulin levels, among other advantages [3,4,5,6,7,8].

Regardless of the age or weight of the child, breast milk is the best food a baby can take even in cases of premature infants [4,9]. However, there are situations in which breastfeeding is contraindicated due to possible allergies, intolerances, low neonatal weight, or personal and/or work reasons [10,11,12], which require its suspension and the establishment of artificial breastfeeding as the only available alternative [13].

Scientific literature has shown that artificial feeding results in faster infant weight gain than breastfeeding; therefore, the combination of both is recommended after the first six months of life [14]. There are different types of artificial feeding formulas: initial or type 1, continuation or type 2, growth or type 3, hypoallergenic [15,16,17].

Among the disadvantages of artificial feeding is the composition of infant formula, as it may contain traces of xenobiotic elements such as fluoride, an electronegative anion found in various foods [18,19,20,21]. Currently, up-to-date studies focused on determining contaminants such as fluoride in infant milk are scarce, although the toxicity of these elements to humans is well described. This contamination can come from the raw material used, such as water, which is a major concern.

Fluorine, the most electronegative halogen element in the periodic table, occurs in nature in ionic form, bound to metallic elements or hydrogen [22,23,24]. The main route of entry into the body is orally, through water, food, dietary supplements and dental products [25]. However, it should be noted that dental products are usually topical and that only accidental ingestions would be referred to. It is absorbed by passive diffusion in 75–90% although this percentage decreases in the presence of certain cations as magnesium (Mg^2+^), calcium (Ca^2+^) and aluminum (Al^3+^), since it tends to form insoluble complexes. For this reason, in formula feeding, absorption decreases by 25% [25].

Fluoride accumulates in calcified tissues, bones and teeth, which is about 2.5 g total body and a concentration ranging from 0.10 to 0.45 mg/L in the blood. The accumulation of fluoride levels in children under 7 years of age can account for approximately 55% of body weight. Its elimination is mostly renal in adults, while in children it only reaches an elimination rate of 45% and in infants 10–20%, the rest is excreted at the intestinal level [22,26,27,28].

Excessive fluoride consumption in children can cause dental fluorosis, a pathological alteration of the forming enamel that makes it porous, with the appearance of white spots that can evolve into a yellow-brown coloration and increase dental fragility. A dose of 0.05–0.07 mg F^−^/Kg/day in children aged 2 to 8 years has been determined to pose a significant risk [27,28,29,30,31].

In addition, exposure to elevated levels of fluoride in drinking water has been associated with a reduction in IQ and cognitive decline in children, suggesting a neurotoxic effect [20,32,33]. Thyroid alterations due to inhibition of iodine absorption have also been described, which may be especially relevant in pregnancy and contribute to congenital hypothyroidism [34,35,36]. Fluoride can cross both the placental barrier and the blood–brain barrier and can reach the fetal brain, presenting teratogenic effects [37,38,39,40,41]. Additionally, fluoride has been suspected of causing damage to female reproductive function [42].

Other adverse effects include kidney damage in children exposed to fluoride concentrations greater than 2 mg/L in drinking water. Prolonged exposure to high levels of fluoride inhibits glycolysis, ATP production and reversibly inhibits insulin secretion. Likewise, bone fluorosis is also one of the effects of chronic exposure to this anion that tends to be deposited on bones [31,43,44,45,46].

These alterations make it necessary to establish reference values. The National Institutes of Health (NIH) establishes reference values of Upper Level (UL), “The largest daily intake of a nutrient considered safe for most people”, for the different age groups of the population, including children of breastfeeding age. According to the NIH, specifically the IOM (Institute of Medicine), the UL values established for infants are: 0.7 mg/day (0–6 months of age), 0.9 mg/day (7–12 months of age) and 1.3 mg/day (1–3 years of age) [47].

Considering the vulnerability of the target population and the ubiquity of fluoride, which may be found in follow-on milk, the objectives of the study are (1) to determine the fluoride content and analyze the possible statistical differences among starter, continuation and hydrolyzed formulas, (2) to evaluate the estimated daily intake (EDI) of fluoride from the consumption of different milk formulas and (3) to assess the toxicological risk associated with the consumption of the analyzed infant formulas according to the age group.

## 2. Material and Methods

### 2.1. Samples

A total of 46 samples of milk preparations were included and stratified according to their characteristics (Table 1). The samples were coded by assigning a letter that replaces the commercial name of each infant formulas. The samples were distributed as follow: Type 1 (No. samples = 18), Type 2 (No. samples = 11), Type 3 (No. samples = 9) and Hydrolysate (No. samples = 8).

The samples were acquired in supermarkets, pharmacies and at the University Hospital of the Canary Islands (Tenerife, Spain). The infant formulas were stored at room temperature, in a dry and safe place, away from environmental contaminants and temperature fluctuations. Once opened, they were immediately analyzed.

The analyzed samples are commercially classified into the following categories [1,17]:

- Starter or Type 1 formulas: Used from birth to the fourth or sixth month of the infant’s life. Their composition aims to mimic that of breast milk and must fully cover the infant’s nutritional needs.
- Follow-on or Type 2 formulas: Used from the fourth or sixth month of the infant’s life. This type of milk does not provide enough energy to cover the metabolic needs of the newborn, so complementary feeding must be given. About 500 mL per day is recommended, providing about half of the daily dietary intake.
- Growing-up or Type 3 formulas: These are dairy products offered as a transition between follow-on milk (Type 2, for babies 6 to 12 months) and cow’s milk. Their composition is adapted to the nutritional requirements of children at this stage of growth, which differ from those of younger infants.
- Hypoallergenic FH (protein hydrolysate) formulas: These formulas are included among the special types. They contain predigested proteins that do not require enzymatic hydrolysis by the pancreas and are more easily absorbed in the proximal small intestine. They are produced from milk formulas treated with heat, enzymatic hydrolysis, and ultrafiltration to remove higher-molecular-weight peptides, thereby reducing antigenicity. Some infant formulas are also based on synthetic amino acids with no antigenic load. Their osmolarity is usually much higher than that of conventional formulas, and they are primarily indicated for cases in which absorption is compromised, digestive diseases with fat malabsorption, or allergy to cow’s milk proteins.
- Lactose-free milk: Also included among special formulas. In these, lactose is replaced by other carbohydrates such as dextrin maltose, glucose polymers, sucrose or glucose. Their main indication is for infants with congenital or acquired lactase deficiency or with galactosemia.

### 2.2. Sample Treatment and Fluoride Determination

To avoid potential interactions of fluoride with borosilicate in the glassware, plastic material was used throughout the study. Ultrapure Milli-Q distilled water was obtained from an ultrafiltration system (Milli-Q^®^ EQ 7000, Merck Millipore, Merck KGaA, Darmstadt, Germany).

A 10^−1^ M fluoride stock solution was prepared by dissolving 4.428 g of NaF (Sigma Aldrich, Schnelldorf, Germany), previously dried in an oven (Nabertherm, Lilienthal, Germany) at 120 °C for 2 h, in 1 L of Milli-Q quality distilled water. To eliminate potential interferences during determination, a conditioning solution of orthophosphoric acid (H_3_PO_4_) 0.75 M from 85% orthophosphoric acid (Sigma Aldrich, Steinheim, Germany) was prepared and brought to a total volume of 1 L with Milli-Q quality distilled water.

Before potentiometric determination of the samples, a calibration curve was prepared from the fluoride stock solution, with decreasing concentrations from 10^−1^ M to 10^−5^ M. The 0.75 M orthophosphoric acid conditioning solution was added to all serial dilutions [23,24,48].

**Figure 1:**
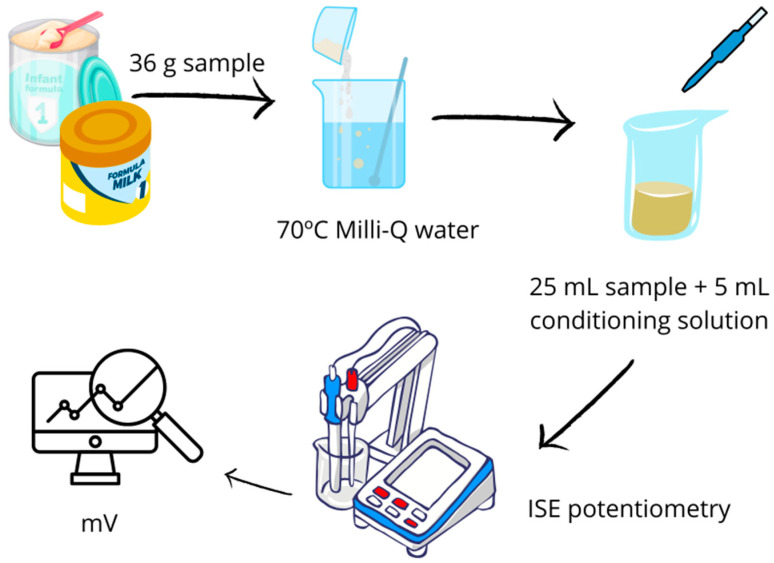
Diagram of the determination of fluoride in the samples.

Samples that were not ready-to-use required prior preparation. This consisted of dissolving 36 g of the follow-on formula in 100 mL of previously heated Mili-Q water (70 °C). The potential (mV) of each sample was then measured using 25 mL of the prepared sample and 5 mL of conditioning solution (Figure 1).

The fluoride determination was determined using a fluoride Ion Selective Electrode (EWI) (HACH LZ55C.97.002F, HACH, Düsseldorf, Germany) and a potentiometer (HACH SensION-MM340, HACH, Düsseldorf, Germany) [49]. Instrument parameters were as follows: (0.01 to 19,000 mg/L), pH range (4 to 8), linear range (0.1 to 19,000 mg/L), slope (59 mV/pF), and operating temperature (5–50 °C).

### 2.3. Quality and Validation of the Method

Quality control of the analytical method was based on an evaluation of its accuracy under reproducibility conditions using the standard addition method [50]. After determining the inherent fluoride concentration in the samples, a known amount of analyte was added. Repeatability studies yielded a relative standard deviation (RSD) of 2.20%, while reproducibility was 3.15%. The recovery percentages obtained were 95%, with an RSD below 10%. Based on these results, the method’s accuracy was considered satisfactory.

### 2.4. Dietary Intake Assessment

The risk assessment of fluoride intake was based on calculating the estimated amount of fluoride ingested from a bottle. Since bottle sizes vary, a 90 mL bottle was used as the reference portion, with consumption scenario of 1, 3 and 7 servings per day. Both the estimated daily intake (EDI) and the percentage of contribution to the UL (%) were obtained using the following equations [51,52]:

$$EDI\left(\frac{mg}{day}\right)=Consumption\left(\frac{L}{day}\right)\times Fluoride\;concentration\;(\frac{mg}{L})$$

$$Contribution\;percentage\;\left(\%\right)=\frac{EDI}{UL}\times 100$$

The UL reference values used were: 0.7 mg/day (0–6 months of age), 0.9 mg/day (7–12 months of age), and 1.3 mg/day (1–3 years of age) [47].

### 2.5. Statistical Analysis

A statistical analysis was conducted to determine the existence of significant differences (*p* < 0.05) among the different types of samples analyzed (Type 1, Type 2, Type 3, and hydrolyzed formulas) and according to their format (powdered or ready-to-use). The GraphPad Prism program (version 10.0, GraphPad Software, Boston, MA, USA) was used. The data distribution was first tested for normality using the D’Agostino&Pearson, Anderson-Darling, Shapiro–Wilk and Kolmogorov–Smirnov tests. Since the data did not follow a normal distribution, non-parametric tests were applied to assess significant differences using the Mann–Whitney two-tailed test for independent variables [53,54,55,56,57,58]. Values of *p* < 0.05 were considered statistically significant.

## 3. Results and Discussion

### 3.1. Fluoride Concentration in the Samples Tested

Table 2 shows the average fluoride concentration (mg/L) by type of follow-on milk and by presentation (powdered or ready-to-use).

The overall mean fluoride of the samples analyzed was 1.13 ± 0.90 mg/L. Almost 75% of the sample means (73.9%) registered concentrations below 1 mg/L. However, 13.0% of the samples analyzed registered values between 1–1.5 mg/L, another 13.0% of the samples analyzed exceeded 1.5 mg/L of fluoride.

Regarding the classification by formula type, hydrolyzed milk exhibited the highest mean concentration (3.37 mg/L), with a maximum value of 7.32 mg/L. In contrast, the lowest mean concentration was found in Type 3 follow-on milks (0.47 mg/L). When classified by presentation format, ready-to-eat formulas showed the highest mean concentration (1.54 mg/L), with a maximum of 9.02 mg/L.

The statistical analysis revealed significant differences among formula types:

Type 1 vs. Type 2 (*p* = 0.0066), Type 1 vs. Type 3 (*p* < 0.0001), Type 1 vs. Hydrolyzed (*p* < 0.0001), Type 2 vs. Type 3 (*p* = 0.0172), Type 2 vs. Hydrolyzed (*p* < 0.0001), and Type 3 vs. Hydrolyzed (*p* < 0.0001). These differences may be attributed to the presence of different ingredients, additives, or the raw materials used, as each formula type corresponds to distinct age groups and nutritional requirements.

When comparing samples by presentation format, no significant differences were found between powdered and ready-to-use formulas (*p* = 0.8594). Therefore, it can be deduced that the format does not influence the fluoride concentration in the analyzed samples.

### 3.2. Comparison of Determined Fluoride Content with Labeling

Some analyzed samples indicated the fluoride content of the product on their labels. Table 3 shows the values declared by the manufacturers compared with those obtained in this study (Table 3).

When comparing declared and measured concentrations, approximately 95% of the samples showed higher fluoride levels than those stated on their labels (positive discrepancy), while 4.8% recorded lower values (negative discrepancy). These differences are remarkable; however, they must be addressed from the perspective established in the legislation µg F/100 kcal.

According to the legislation governing these products, the composition and labeling of infant and follow-on formulas are regulated generally by Regulation (EU) No 609/2013 and specifically by Regulation (EU) 2016/127 [59,60]. These regulations establish a maximum fluoride content of 100 µg F/100 kcal.

**Figure 2:**
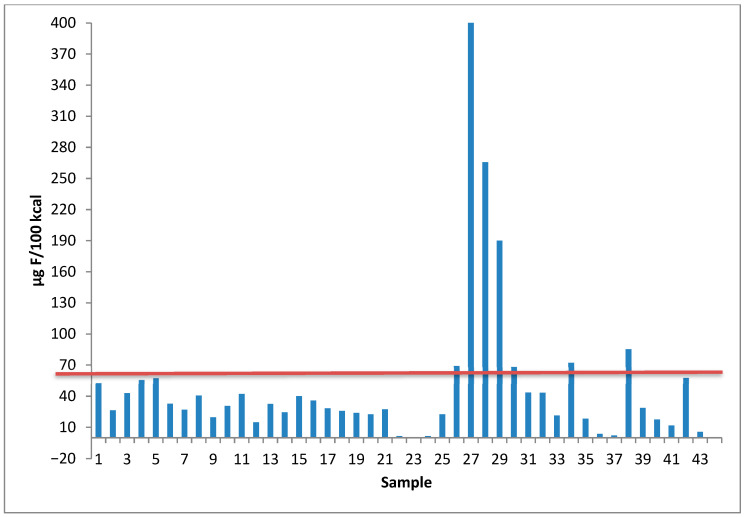
Fluoride content expressed in 100 kcal of sample and comparison with the maximum permissible value of 100 µg/100 kcal (red line).

As shown in Figure 2, three analyzed samples exceeded this limit, all belonging to hydrolyzed follow-on formulas. Notably, these three samples were from different brands, and two other hydrolyzed formulas were found to be close to the legal limit.

### 3.3. Comparison with Other Authors

Table 4 presents a comparison of fluoride concentrations in infant formulas reported by other authors. A 1993 study analyzing follow-on milk from Japan and Brazil for infants up to six months of age reported fluoride concentrations ranging from 0.53 to 1.33 mg/L [61]. These values are higher than the average concentrations recorded in this study for Type 2 and Type 3 formulas, although one Type 3 brand in this study reached a maximum of 1.75 mg/L, exceeding those earlier findings. This indicates that, despite more than two decades between studies, disparities in fluoride concentrations persist, with some brands still showing high levels that may pose a risk.

Bussell et al. (2016) [62], analyzing samples marketed in the United Kingdom for infants aged 0–24 months, found concentrations between 0.002 and 0.282 mg/L, with a mean of 0.025 mg/L. They also reported significant differences between hospital-based formulas (higher concentrations) and those freely marketed [62].

A more recent study from the United Arab Emirates [63] analyzed follow-on milk formulas (6–12 months) from both the EU and non-EU regions, reporting concentrations between 0 and 0.4 mg/L, with an average of 0.19 mg/L. Other studies published in 2022 and 2023 show the same trend [5,63,64]. These results are lower than those obtained in the present study, again confirming a lack of standardization in infant formula production.

Overall, the present study found a much wider range of fluoride concentrations (0.004–9.02 mg/L), highlighting significant variability across brands and formula types, with some products exceeding safe limits.

**Table 4:** Comparison with other authors.

Reference	Conc. Fluoride (mg/L)	Age of the Population (Months)
[59]	0.53–1.33	6
[65]	0.05–0.28	<24
[66]	0.031–0.532	1 to 12
[67]	0.01–0.75	Unknown
[62]	0.002–0.282	0–24
[63]	0–0.4	6–12
[68]	0.009–0.197	0–12
[64]	0.003–0.035 (liquid formulas)	0–12
	0.010–1.2 (solid formular)	
[69]	0.01–0.92 (milk-based formulas)	0–12
	0.13–1.11 (soy-based formulas)	
[5]	0.031–0.07	0–12
Present study, 2025	0.004–8.09 (poder)	0–12
	0.03–9.02 (ready for consumption)	

### 3.4. Dietary Intake Assessment and Risk Characterization

Dietary intake assessment was performed by calculating the estimated daily intake (EDI) for the lowest, average, and highest concentrations found for each type of formula. Thereafter, risk characterization was performed based on the UL values established by the IOM (Table 5). These values were calculated for an estimated consumption of 1 bottle, 3 bottles and 7 bottles of 90 mL each, as most manufacturers’ consumption recommendations vary the number of bottles per day rather than the amount of formula per bottle.

In the case of the consumption of formulas for Type 1 infants, it is necessary that 1 serving of 90 mL would mean a contribution percentage to the UL of 3.43% to 19.9% for infants (0–6 months of age). These values are notoriously lower than the tolerable value, so there would be no risk for this age group, if we consider only the consumption of this product. It is necessary to consider the contribution of the water with which the bottle is prepared. In the case of children aged 7–12 months (2.67% to 15.4% of the UL) and between 1–3 years (1.85% to 10.7% of the UL), the risk is even lower.

In the case of the consumption of Type 2 infant formulas, for all age groups, the contribution percentages remain below 12.7%, indicating that there would be no risk from the consumption of 1 serving of 90 mL of Type 2 formulas. While, in the case of Type 3 formulas, in the case of children from 7 to 12 months of age, it is considered a significant contribution of the brand with the highest concentration registered, offering a contribution percentage of 22.6% of the UL.

However, hydrolyzed formulas can pose a risk to health, if we consider the contribution from the brand that registers the maximum value in this group, offering a contribution percentage of 94.1% of the UL with only 1 serving of 90 mL in the age group of 0–6 months. Likewise, the risk is repeated in the age group of 7 to 12 months and in the group of 1 to 3 years of age, due to the same brand (73.2% and 50.7% of the UL, respectively). In these cases, and assuming that the water with which the bottles are prepared may contain fluoride, there would be a risk of high intake of this toxic anion.

As the number of daily servings increases, the risk of high fluoride intake is focused on the consumption of the brand’s hydrolysate formulas that registered the highest average concentration. The product “Hydrolyzed” presents a very high and dangerous risk of exceeding the tolerable upper limit for infants (0–6 months, 7–12 months) and children (1–3 years) at the highest intake levels. The %UL values in the tens of thousands are alarming and indicate a significant safety concern.

It is therefore recommended that a regulatory framework be established to control fluoride concentrations in infant formulas and require accurate labeling of fluoride content for all formula types (Type 1, Type 2, Type 3, and hydrolyzed). In approximately 41.3% of the analyzed samples, manufacturers did not specify fluoride concentrations. Additionally, parents and caregivers should be informed about the fluoride content of the tap or bottled water used for formula preparation, as this can significantly increase total fluoride intake and consequently the risk to infant health.

## 4. Conclusions

Analysis of the different milk formulas indicates that hydrolyzed formulas have the highest fluoride concentrations, while Type 3 formulas contain the lowest amounts. From a safety standpoint, the daily consumption of one bottle of any milk formula type is considered safe for all age groups. When intake increases to three bottles per day, Type 1, Type 2, and Type 3 formulas remain safe across all age groups. However, some commercial brands of hydrolyzed formulas may pose a health risk, particularly for infants aged 0–6 months and 7–12 months, due to their higher fluoride content. Consumption of seven bottles per day may also represent a potential risk for infants consuming Type 1 and Type 3 formulas with higher fluoride concentrations. In the case of hydrolyzed formulas, such intake levels could pose a health risk across all age groups.

Based on the results of this study, it is recommended that further research be conducted on the influence of the type of water used in formula preparation, as it appears to be a determining factor in total fluoride concentration. In addition, more comprehensive studies on actual dietary fluoride intake and an expanded food composition database—including fluoride content in various children’s products—are needed.

## Figures and Tables

**Table 1:** Samples and classification by type, brand and production.

Sample	Brand	Type	Sample	Brand	Type
1	I	Type 1	22	I	Type 3
2	M		23		
3	L		24		
4	F		25	B	
5	E		40	L	
6	F		43	N	
7	L		44		
8	C		45		
9	I		46		
10			27	B	Hydrolyzed
11			28	K	
12	D		29	I	
21	H		30		
32	A		31	B	
33	K		35	I	
34	G		36	M	
38	B		37	I	
42	J				
13	I	Type 2			
14					
15	L				
16	F				
17	D				
18	B				
19	I				
20	I				
26	E				
39	D				
41	A				

The production method for all samples is conventional. The samples 1–21; 25–34; 38–42 are presented in powder format and the samples 22–24; 35–37 and 43–46 in ready/ready for consumption format.

**Table 2:** Mean concentrations (mg/L) and standard deviations (SD) of the samples analyzed according to types and presentation format.

	Fluoride Conc. (mg/L) ± SD	Min.–Max. Value
**Type**		
Type 1	0.75 ± 0.35	0.27–1.54
Type 2	0.53 ± 0.22	0.16–0.99
Type 3	0.47 ± 0.58	0.03–1.75
Hydrolyzed	3.38 ± 2.78	0.77–7.32
**Presentation format**		
Powder	1.02 ± 1.35	0.004–8.09
Ready for consumption	1.54 ± 2.36	0.03–9.02

**Table 3:** Concentration declared on the label and comparison with the fluoride content obtained in the present study.

Sample	Fluoride Conc. (mg/100 mL)	Declared Value (mg/100 mL)	Difference (%)
1	0.094	0.01	840.00
2	0.046	0.006	666.67
3	0.078	0.01	680.00
4	0.107	0.00069	15,407.25
6	0.055	0.00066	8233.33
7	0.048	0.01	380.00
8	0.074	0.0065	1038.46
9	0.037	0.01	270.00
10	0.057	0.01	470.00
11	0.078	0.000079	98,634.18
13	0.059	0.008	637.50
14	0.044	0.01	340.00
15	0.068	0.01	580.00
16	0.069	0.00069	9900.00
18	0.045	0.000371	12,029.38
20	0.04	0.01	300.00
25	0.038	0.00069	5407.25
28	0.476	0.06	693.33
29	0.333	0.21	58.57
30	0.124	1	−87.60
32	0.075	0.006	1150.00
33	0.039	0.06	−35.00
34	0.127	0.001	12,600.00
38	0.154	0.000358	42,916.76
41	0.016	0.006	166.67
42	0.107	0.01	970.00

**Table 5:** EDI values and percentage of contribution to the UL according to different consumption scenarios and calculated for the lower, average value and upper value of fluoride obtained in each type of product.

Product Type	1 Serving	% UL			3 Servings	% UL			7 Servings	% UL		
		Infants		Children		Infants		Children		Infants		Children
	EDI (mg/Day)	0–6 Months	7–12 Months	1–3 Years	EDI (mg/Day)	0–6 Months	7–12 Months	1–3 Years	EDI (mg/Day)	0–6 Months	7–12 Months	1–3 Years
Type 1	0.02	3.43	2.67	1.85	0.07	10.4	8.11	5.62	0.17	24.3	18.9	13.1
	0.07	9.71	7.56	5.23	0.20	29.0	22.6	15.6	0.47	67.6	52.6	36.4
	0.14	19.9	15.4	10.7	0.42	59.4	46.2	32.0	0.97	139	108	74.6
Type 2	0.01	2.00	1.56	1.08	0.04	6.14	4.78	3.31	0.10	14.4	11.2	7.77
	0.05	7.00	5.44	3.77	0.15	20.9	16.2	11.2	0.34	48.6	37.8	26.2
	0.09	12.7	9.89	6.85	0.27	38.1	29.7	20.5	0.62	89.1	69.3	48.0
Type 3	0.00	0.43	0.33	0.23	0.01	1.14	0.89	0.62	0.02	2.71	2.11	1.46
	0.04	6.14	4.78	3.31	0.13	18.6	14.4	10.0	0.30	43.1	33.6	23.2
	0.16	22.6	17.6	12.2	0.47	67.6	52.6	36.4	1.12	158.2	123.2	85.4
Hydrolyzed	0.07	9.86	7.67	5.31	0.21	29.7	23.1	16.0	0.49	69.3	53.9	37.3
	0.30	43.4	33.8	23.4	0.91	130	101	70.2	2.1	303.8	236.6	163.8
	0.66	94.1	73.2	50.7	1.98	282.3	219.6	152.1	4.62	658.7	512.4	354.9

## Data Availability

The original contributions presented in this study are included in the article. Further inquiries can be directed to the corresponding author.
